# Supplementary material for: The role of ion dissolution in metal and metal oxide surface inactivation of SARS-CoV-2
Source: Appl Environ Microbiol. Author manuscript; Available in PMC 2024 Mar 8. (PMC10880620; doi:10.1128/aem.01553-23)
Supplement: Supplementary material [file EMS192997-supplement-Supplementary_material.pdf]

Supplementary Material:

The role of ion dissolution in metal and metal oxide surface inactivation of SARS-CoV-2

Jane Hilton[a*], Yoshiko Nanao[b*], Machiel Flokstra[b], Meisam Askari[b$], Terry K. Smith[a] Andrea Di Falco[b] Phil D.C. King[b], Peter Wahl[b#], Catherine S Adamson[a#]

[a] Biomedical Sciences Research Complex, School of Biology, University of St Andrews, St Andrews, Fife, UK

[b] SUPA, School of Physics and Astronomy, University of St Andrews, St Andrews, Fife, UK

Running Head: Surface Inactivation of SARS-CoV-2

[#]Address correspondence to Catherine S Adamson, csa21@st-andrews.ac.uk or Peter Wahl, gpw2@st-andrews.ac.uk

* Jane Hilton and Yoshiko Nanao contributed equally to this work. Author order was determined as Jane Hilton contributed the biological data, presented in the paper, whereas Yoshiko Nanao generated the test surfaces used in the study.

[$] Present Address: Optek Systems, Abingdon, Oxford, UK

Table S1 Summary of surfaces tested in this study as well as substrate materials, growth methods and profiles, and thickness.

Tested Surfaces	Substrate	Growth method	Growth profile	Thickness (nm)
Evaporated Copper (EC)	NiCr/Si	E-beam	Surface was deposited with e-beam in vacuum at room temperature after adhesive layer (Ni-Cr) deposition on Si.	0 – 500
Silver (Ag)	NiCr/Si	E-beam		250
Bismuth (Bi)	NiCr/Si	E-beam		270
Nickel (Ni)	Glass	MBE	Elemental metal sources were thermally evaporated in vacuum at room temperature directly on glass.	30
Palladium (Pd)	Glass	MBE		20
Copper Chromate ($CuCrO_2$)	Al_2O_3	MBE	Cu and Cr was evaporated alternatively. O_2 pressure and substrate temperature was kept at 5 x 10^{-6} mbar and at 800 ^oC, respectively.	25
Indium Tin Oxide (ITO)	Glass	RF sputtering	ITO was sputtered at 200 ^oC with the total pressure of 3 mTorr, followed by post annealing for 30 min.	10
Titanium Oxide (TiO_2)	Glass	MBE	Ti was evaporated from effusion cell while O_2 pressure and substrate temperature were kept at 5 x 10^{-6} mbar and at 700 ^oC, respectively.	16
Annealed Evaporated Copper (CuO/Cu_2O) mixture	NiCr/Si	Annealing in air	Evaporated copper films were rinsed with acetone and 2-propanol for 5 min each, and dried at 200 ^oC for 5 min, then placed on hot surface at 350 ^oC in air and left for 1 hour. Cooled in air down to 200 ^oC then removed from the hot surface. Films were treated with water when required.	100
				500
Copper Oxide (Cu_2O)	Glass<br>LSAT	MBE	Cu was evaporated at 650 ^oC in 10 % O_3 environment where total pressure was kept at approx. 2 x 10^{-5} mbar.	10
				30

Table S2 Summary of cutting, processing and storage of the surfaces tested in this study.

Tested Surfaces	Cutting	Pre-processing	Storing after deposition
Evaporated copper (EC)	Manual cut with diamond pen after deposition	Rinsed with acetone then 2-propanol in an ultra sonicator	Air
Silver (Ag)	(as above)	(as above)	(as above)
Bismuth (Bi)	(as above)	(as above)	(as above)
Nickel (Ni)	Precut substrates were used	(as above)	(as above)
Palladium (Pd)	(as above)	(as above)	(as above)
Copper Chromate ($CuCrO_2$)	(as above)	(as above)	N_2 desiccator
Indium Tin Oxide (ITO)	Manual cut with diamond pen after deposition	(as above)	Air
Titanium Oxide (TiO_2)	Precut substrates were used	(as above)	(as above)
Annealed Evaporated Copper (CuO/Cu_2O) mixture	Cut EC coupons were used	Rinsed with water when needed	Vacuum storage
Copper Oxide (Cu_2O)	Precut substrates were used	Rinsed with acetone then 2-propanol in an ultra sonicator	(as above)
Copper foil	Cut with metal cutter	Polished one side, followed by rinsing with acetone, 2-propanol in an ultra sonicator	Air
Stainless steel	(as above)	(as above)	(as above)

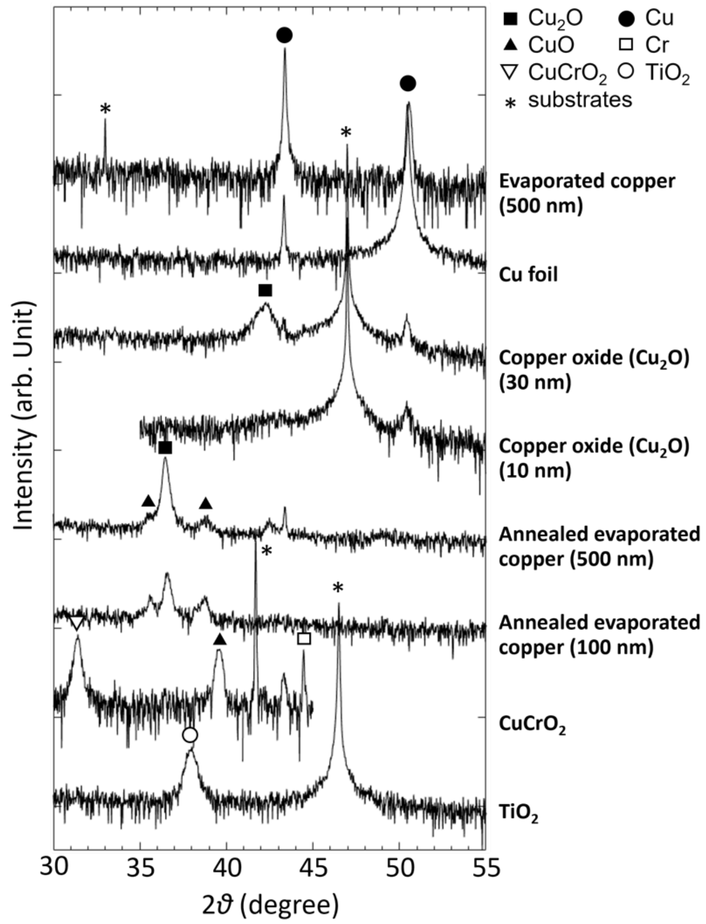

FIG S1 XRD patterns of surfaces tested in this study. Si (1 0 0) with adhesive layer of Ni-Cr alloy was used as a substrate material for films of evaporated copper and annealed copper, while Al_2O_3 (0 0 0 1), $(LaAlO_3)_{0.3}(Sr_2TaAlO_6)_{0.7}$ (LSAT) (0 0 1), and $SrTiO_3$ (0 0 1) were used for stabilising $CuCrO_2$ and binary oxides, respectively. Diffraction peaks from substrate materials are all shown with asterisks (*). Note that the samples of copper oxide and titanium oxide used for virological tests were grown on glass substrates.

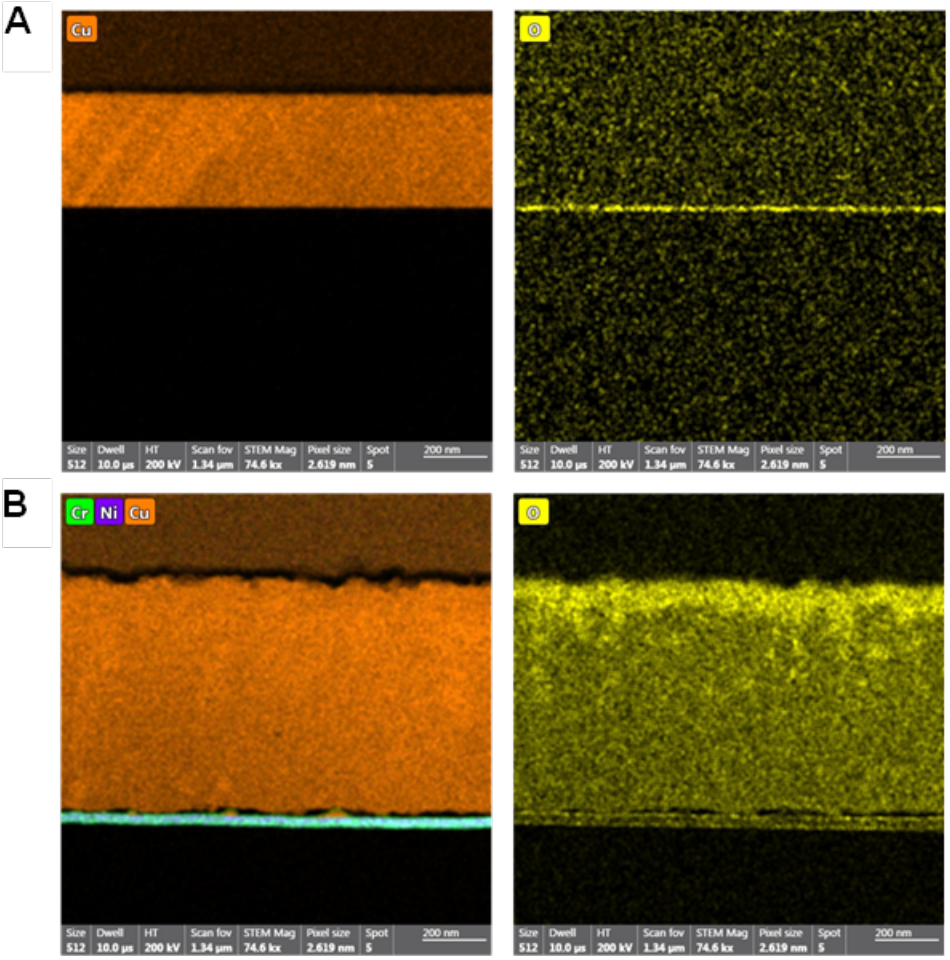

FIG S2 Cross-sectional energy dispersive X-ray analysis images on (A) evaporated copper film on crystalline Si, and (B) annealed evaporated copper. Smooth surface with sharp interface is apparent in images from evaporated copper while the evaporated copper film show rougher surface. Notably, the distribution of oxygen atoms in the annealed copper film (bottom right) is not uniform and higher density of oxygen near the film surface can be seen.

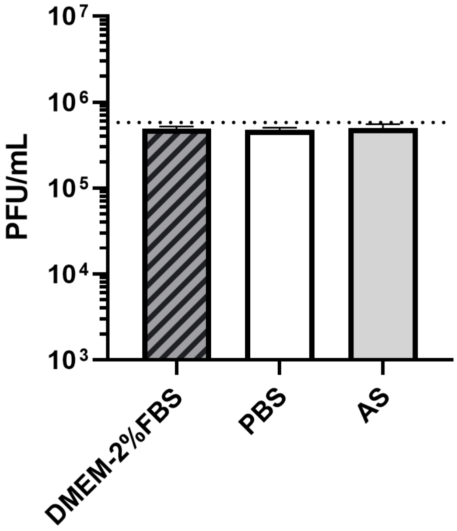

FIG S3 Titre of SARS-CoV-2 in Carrier solutions. Following resuspension of SARS-CoV-2 in each carrier solution, the virus was confirmed as remaining viable. SARS-CoV-2 viral titre was determined, and data is presented as (PFU/mL) in each carrier solution, DMEM-2%FBS, PBS and AS. Data shown represents mean values (n = 3 replicates and error bar = SD) and is representative of 3 independent experiments.

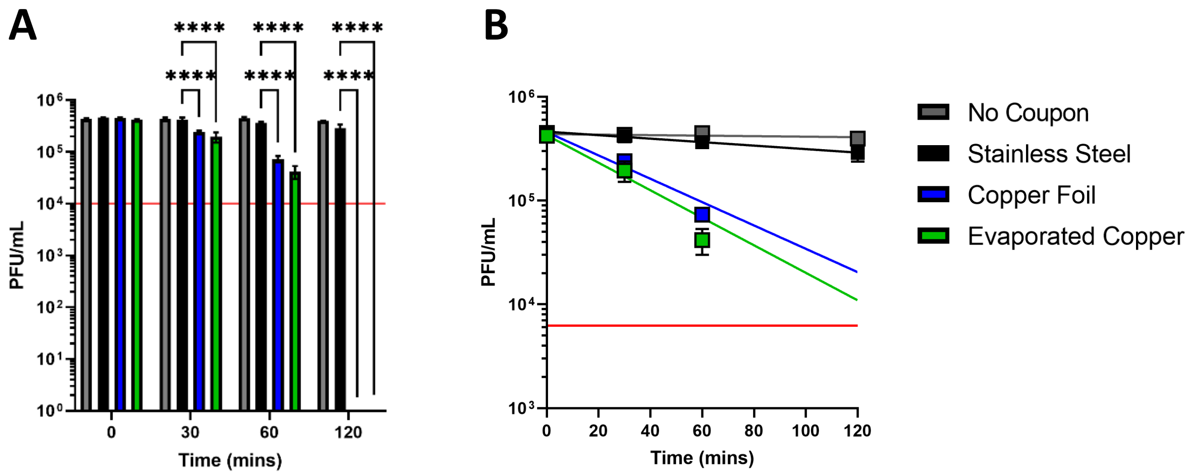

FIG S4 SARS-CoV-2 inactivation upon exposure to copper surfaces over time. (A) Titre of SARS-CoV-2 (PFU/mL) exposed to different metal surfaces after 0-, 30-, 60-and 120-min. Data shown represents mean values (n = 3 replicates and error bar = SD) and is representative of 3 independent experiments. Statistical significance was assessed using two-way ANOVA with Tukeys multiple comparison test, **** $p < 0.0001$. The limit of detection (LOD) for the assay is indicated by the solid red line. (B) Titre of SARS-CoV-2 (PFU/mL) exposed to different test surfaces as a function of time, exponential fits to the data are shown along with a solid red line, which indicates the LOD for the assay.

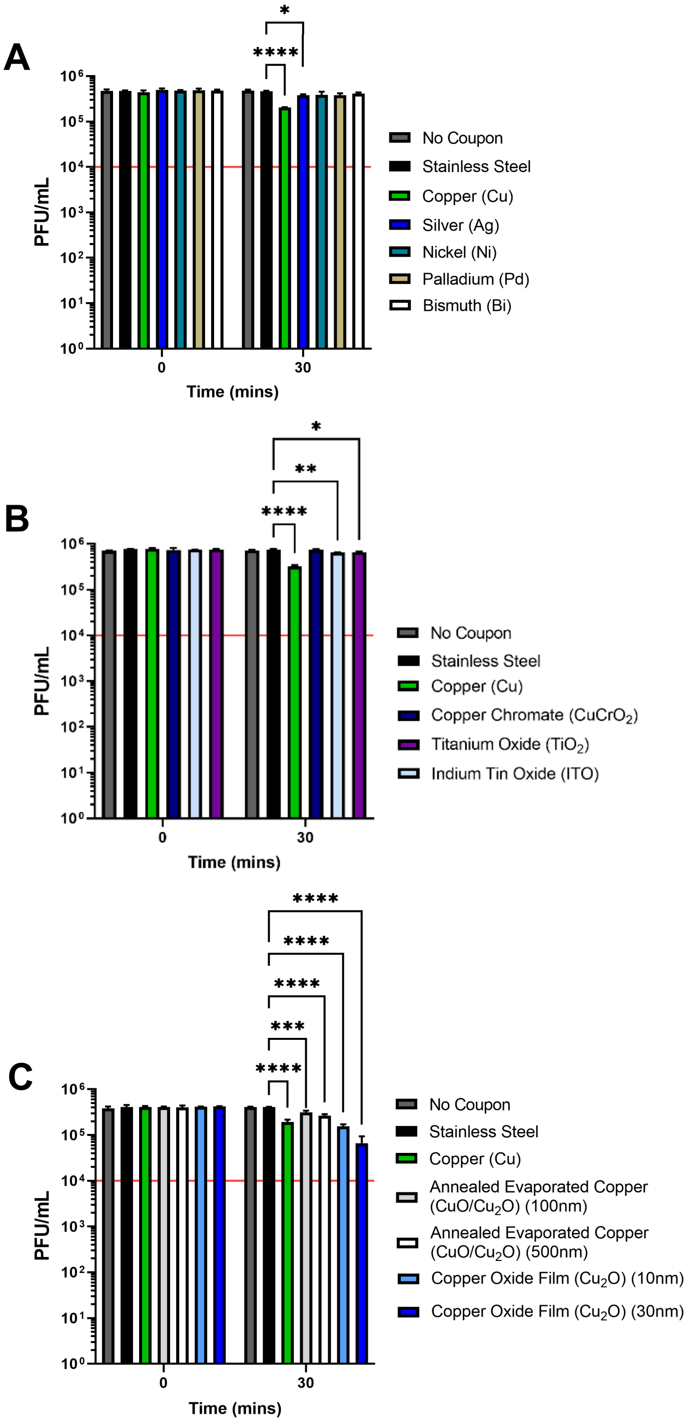

FIG S5 Screening test elemental metal and metal oxide surfaces for SARS-CoV-2 antiviral activity superior to copper. Titre of SARS-CoV-2 (PFU/mL) exposed to different test metal and metal oxide surfaces after 0 and 30 min compared to no coupon, stainless steel, and copper controls. (A) elemental metal test surfaces; silver (Ag), nickel (Ni), palladium (Pd), bismuth (Bi) (B) metal oxide test surfaces; copper chromate ($CuCrO_2$),

titanium oxide (TiO_2), indium tin oxide (ITO) and (C) copper oxide test surfaces; annealed evaporated copper (CuO/Cu_2O mixture) and predominantly Cu_2O containing surfaces, generated at indicated thicknesses. Data shown represents mean values ($n = 3$ replicates and error bar = SD) and is representative of 3 independent experiments. Statistical significance was assessed using two-way ANOVA with Tukeys multiple comparison test, **** $p < 0.0001$,*** $p < 0.001$, ** $p < 0.01$, * $p < 0.1$. The limit of detection (LOD) for the assay is indicated by the solid red line.

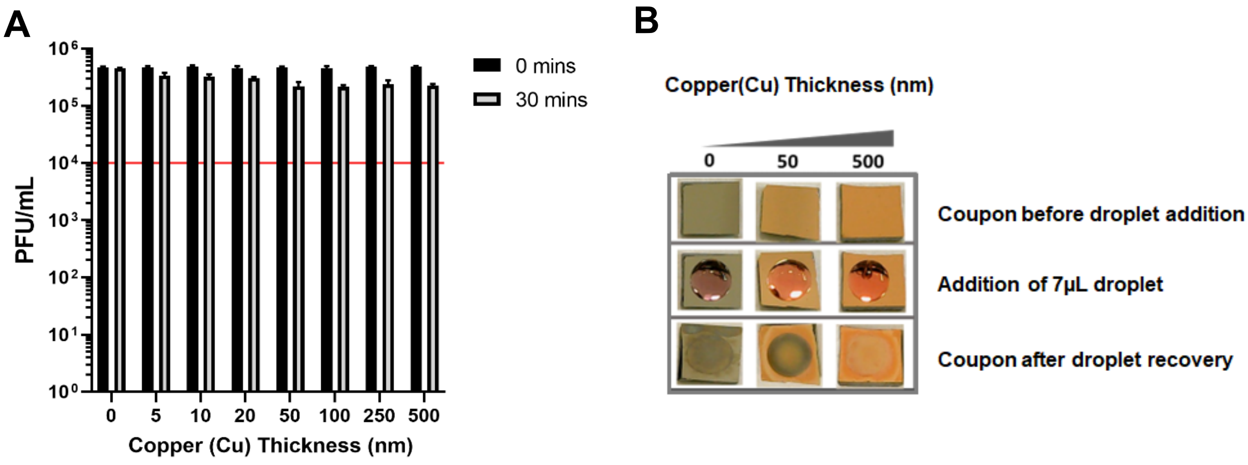

FIG S6 Effect of copper surface film thickness on SARS-CoV-2 inactivation. (A) Titre of SARS-CoV-2 (PFU/mL) exposed to coupons with evaporated copper film of increasing thickness. Data shown represents mean values ($n = 3$ replicates and error bar = SD) and is representative of 3 independent experiments. The limit of detection (LOD) for the assay is indicated by the solid red line. (B) images of evaporated copper thin-film coupons of 50 nm and 500 nm thicknesses before, during and after 30 min incubation with a 7 μL droplet of DMEM-2%FBS.

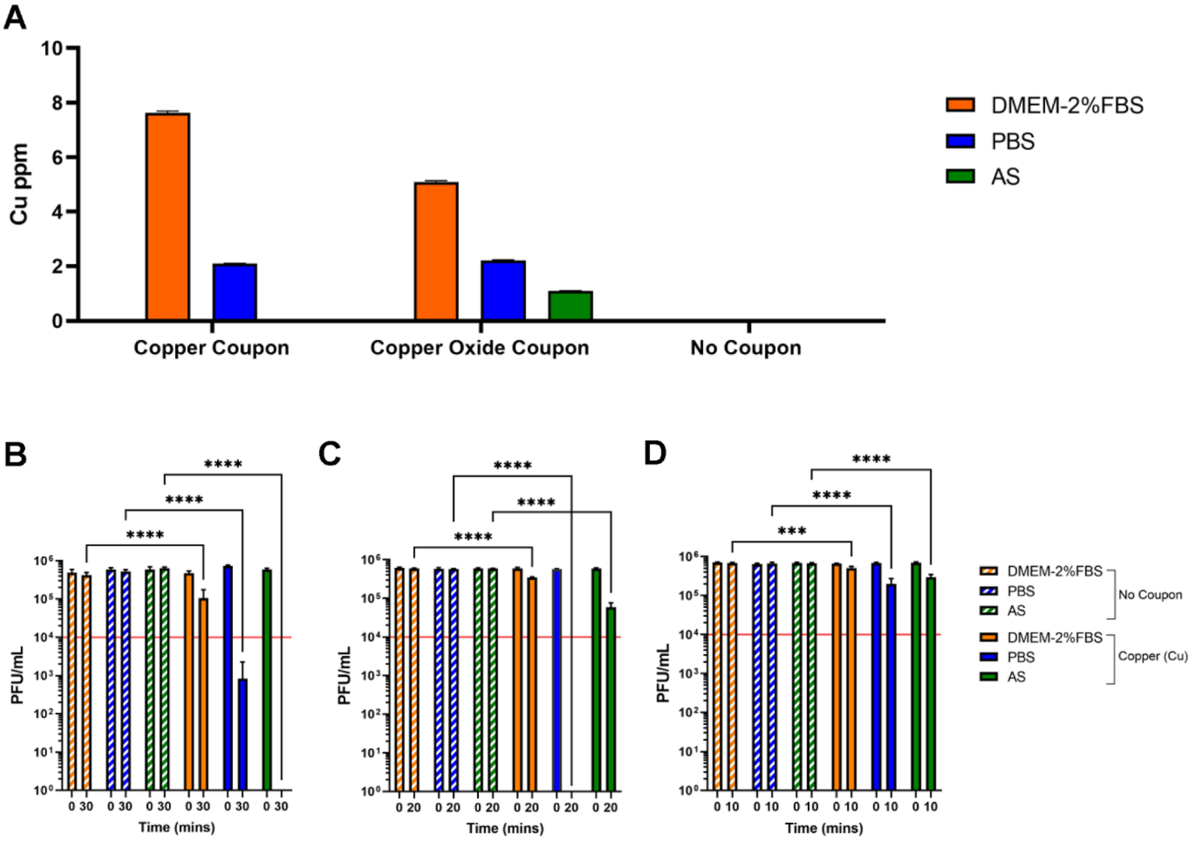

FIG S7 Impact of different carrier solutions on copper ion dissolution and SARS-CoV-2 inactivation upon exposure to an evaporated copper thin-film surface. (A) ICP-OES determined copper ion levels in DMEM-2%FBS, PBS or AS carrier solutions following 30-min exposure to evaporated copper, Cu_2O thin film coupons or no coupon control. Data shown represents mean values (n = 6 replicates and error bar = SD). (B-D) Titre of SARS-CoV-2 (PFU/mL) resuspended in DMEM-2%FBS, PBS or AS carrier solutions and exposed to evaporated copper surfaces for (B) 30, (C) 20 and (D) 10 min or the equivalent no coupon control. Data shown represents mean values (n = 3 replicates and error bar = SD). At the 30 min time point the data shown is representative of 3 independent experiments, the 20- and 10-min time points were included in the 3rd and final

experimental repeat. Statistical significance was assessed using two-way ANOVA with Tukeys multiple comparison test, **** $p < 0.0001$, *** $p < 0.001$. The limit of detection (LOD) for the assay is indicated by the solid red line.

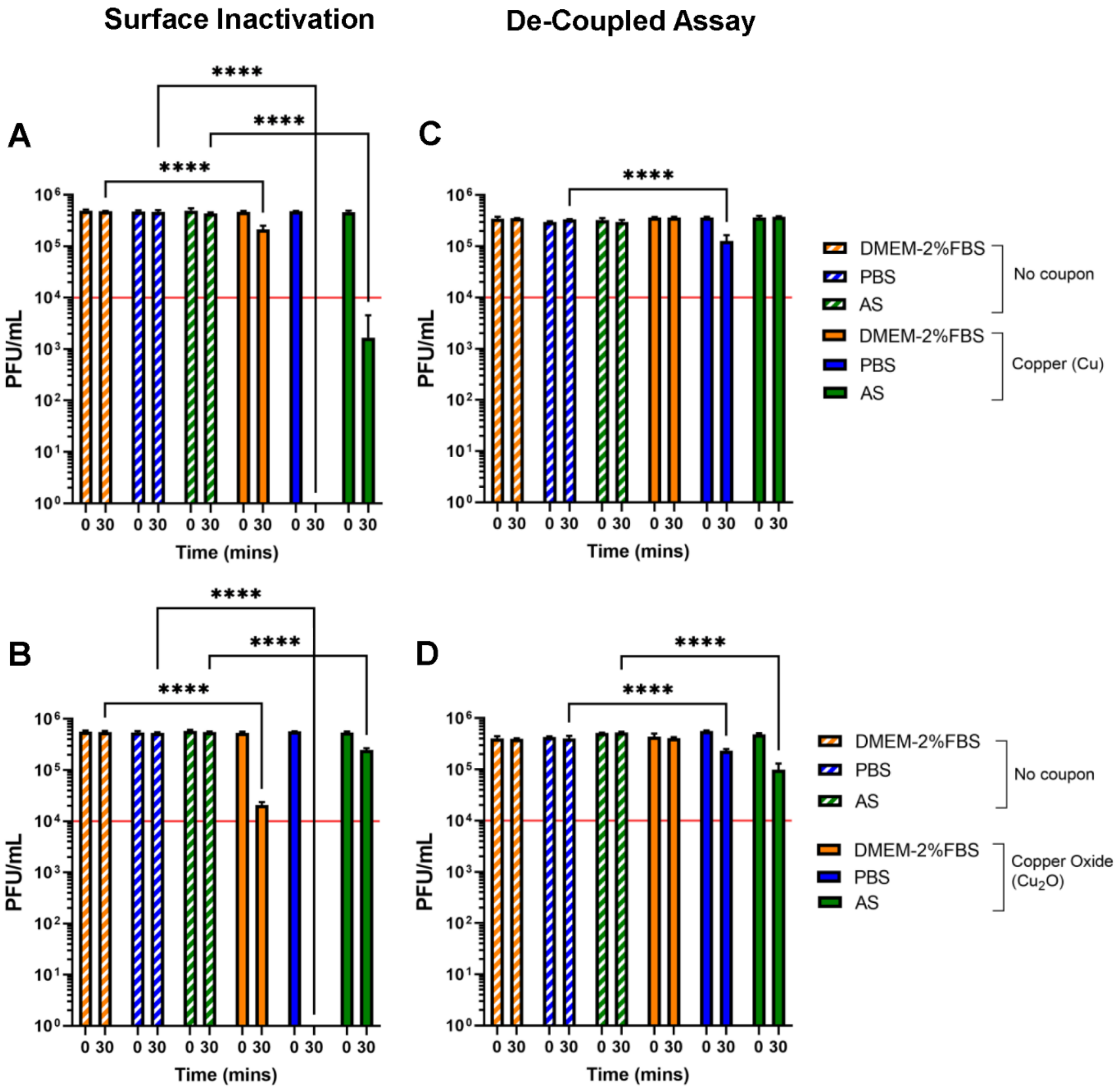

FIG S8 De-coupled ion dissolution SARS-CoV-2 inactivation assay. (A and B) test surface virus inactivation assay: Titre of SARS-CoV-2 (PFU/mL) resuspended in DMEM-2%FBS, PBS or AS carrier solutions and exposed to (A) evaporated copper or (B) Cu_2O thin-film

coupons for 0 or 30 minutes or the equivalent no coupon control. (C and D) de-coupled virus inactivation assay: carrier solution DMEM-2%FBS, PBS or AS exposed to evaporated copper (C) and Cu_2O (D) thin-film coupons for 0 or 30 min or the equivalent no coupon control. Following coupon exposure, the resultant solution is removed and spiked with SARS-CoV-2 and incubated for a further 0 or 30 min or the equivalent no coupon control. Data is shown as titre of SARS-CoV-2 (PFU/mL). Data represents mean values (n = 3 replicates and error bar = SD) and is representative of 3 independent experiments. Statistical significance was assessed using two-way ANOVA with Tukeys multiple comparison test, **** $p < 0.0001$. The limit of detection (LOD) for the assay is indicated by the solid red line.
